# Inference of Cancer-specific Gene Regulatory Networks Using Soft Computing Rules

**DOI:** 10.4137/grsb.s4509

**Published:** 2010-03-24

**Authors:** Xiaosheng Wang, Osamu Gotoh

**Affiliations:** 1 Department of Intelligence Science and Technology, Graduate School of Informatics, Kyoto University, Kyoto 606-8501, Japan; 2 National Institute of Advanced Industrial Science and Technology, Computational Biology Research Center, Tokyo 135-0064, Japan. Email: david@genome.ist.i.kyoto-u.ac.jp

**Keywords:** cancer, microarrays, gene regulatory networks, machine learning, decision rules

## Abstract

Perturbations of gene regulatory networks are essentially responsible for oncogenesis. Therefore, inferring the gene regulatory networks is a key step to overcoming cancer. In this work, we propose a method for inferring directed gene regulatory networks based on soft computing rules, which can identify important cause-effect regulatory relations of gene expression. First, we identify important genes associated with a specific cancer (colon cancer) using a supervised learning approach. Next, we reconstruct the gene regulatory networks by inferring the regulatory relations among the identified genes, and their regulated relations by other genes within the genome. We obtain two meaningful findings. One is that upregulated genes are regulated by more genes than downregulated ones, while downregulated genes regulate more genes than upregulated ones. The other one is that tumor suppressors suppress tumor activators and activate other tumor suppressors strongly, while tumor activators activate other tumor activators and suppress tumor suppressors weakly, indicating the robustness of biological systems. These findings provide valuable insights into the pathogenesis of cancer.

## Background

Although many important genes responsible for the genesis of various cancers have been discovered, the molecular mechanisms underlying oncogenesis remain unclear. Recently, the use of systems biology approaches to understand the disease is generating extensive interest.^1^–^4^ The advent of microarrays has fueled investigations that use whole-genome expression profiles to understand cancer and to identify key cancer-specific gene regulatory networks.^5^–^11^

The construction of gene regulatory networks through microarrays is often called “reverse engineering.” There are two classes of reverse-engineering algorithms: one identifying true physical interactions between regulatory proteins and their promoters, and the other identifying regulatory influences between RNA transcripts.^12^ Here we limit our discussion to the second class: gene-to-gene interaction networks. The interaction between two genes in a gene network does not necessarily imply a physical interaction, but can also refer to an indirect regulation via proteins, metabolites, and ncRNA that have not been measured directly.^13^ In general, there are two classes of gene-to-gene interaction networks: undirected and directed. The popular algorithms for reconstructing undirected networks are based on similarity measures, such as Pearson correlation^14^,^15^ and mutual information,^16^–^18^ to name a few. One obvious deficiency of these methods is that the direction of interaction is not specified. As a result, the cause-effect regulatory relations among genes cannot be well characterized. In contrast, directed gene regulatory networks are capable of depicting the cause-effect regulatory relations, better providing insights into biological systems than the co-expression relation. The oft-used methods for inferring directed networks include Bayesian networks,^19^–^23^ Boolean networks,^24^–^26^ ordinary differential equations (ODEs)^27^–^33^ et al. In the present study, we attempt to develop a method for inferring gene regulatory networks based on soft computing rules,^34^ by which directed regulatory relations between gene pairs can be induced. Although rule-based formalisms have been used for inferring gene regulatory networks by some investigators,^35^–^39^ the use of this kind of methods for inference of gene regulatory networks has not yet been sufficiently explored.

Most of the previous efforts toward the reconstruction of cancer-specific gene networks utilized all gene expression data from microarrays to identify the intricate interplay between genes, some of which actually had nothing to do with the observed cancer phenotype. As a result, gene interactions essentially responsible for oncogenesis were difficult to detect. To better discover authentic gene interactions relevant to cancer, in this work, we reconstruct cancer-specific gene regulatory networks by focusing on a small number of relevant genes, each of which shows good performance in distinguishing cancerous tissues from normal ones. The main objective of this study is to observe the roles played by high class-discrimination genes in the context of cancer-specific gene regulatory networks. We suspect that genes with good classification ability have high centrality in the networks; that is, they are inclined to act as hub genes. We use one colon-cancer-related microarray dataset to validate our suspicion.

## Results and Analysis

We use directed graphs to describe networks, in which each node represents a gene and the presence of a directed edge between two nodes indicates the existence of a regulatory relation between the connected genes. We construct all network graphs using Cytoscape software.^40^ We aim to analyze two classes of networks: one containing only the identified 18 genes (refer to Materials and Methods) (Network Type 1), and the other containing genes other than the 18 genes (Network Type 2). Clearly, the former appears as a subgraph of the latter for identical α values (refer to Materials and Methods).

### Network type 1

**Figure 1.**
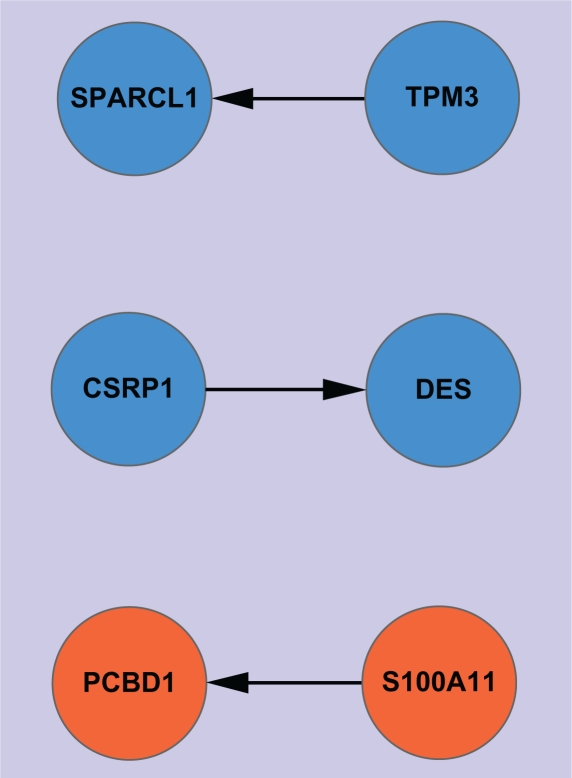
Network Type 1 constructed under α = 0.95.

**Figure 2.**
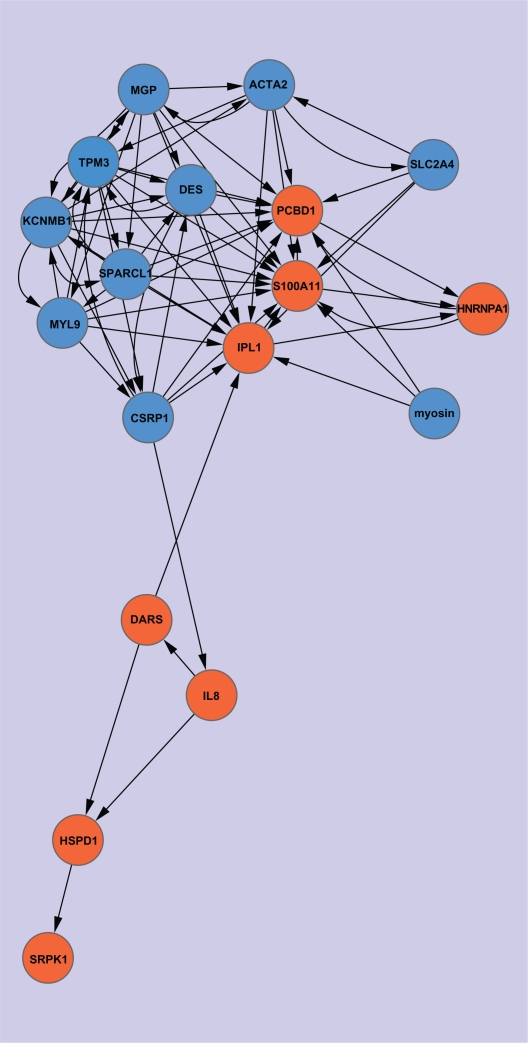
Network Type 1 constructed under α = 0.85.

**Figure 3.**
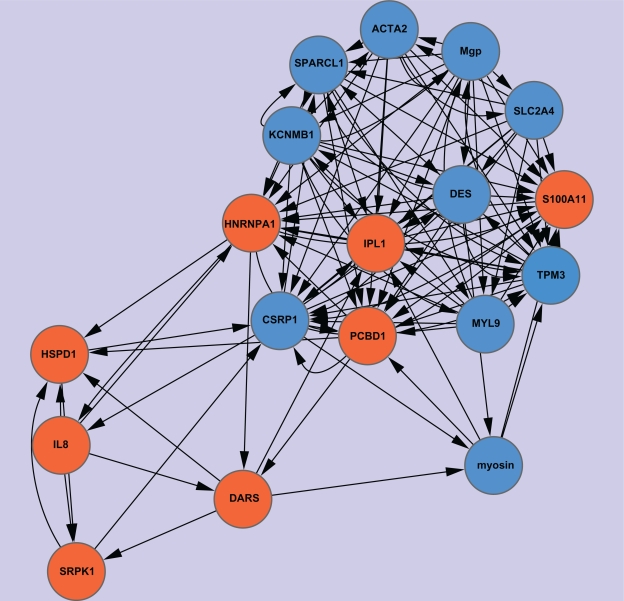
Network Type 1 constructed under α = 0.80.

For Network Type 1, we use red circle nodes to represent upregulated genes in tumor, and blue circle nodes to represent downregulated genes in tumor. Thus, an edge connecting two nodes with identical colors indicates a positive regulatory relation between the two genes. In contrast, an edge connecting two nodes with different colors indicates a negative regulatory relation between the two genes. When *α* = 1, no regulatory relation among the 18 genes is found, and when *α* = 0.95, three regulatory relations are identified (Figure 1). They are TPM3, CSRP1, and S100A11 positively regulating SPARCL1, DES, and PCBD1, respectively. The three regulatory relations are highly reliable because the confidences of all decision rules that infer them are no less than *α* (= 0.95).^34^ The corresponding regulatory networks when α = 0.85 and 0.8 are shown in Figure 2 and Figure 3, respectively. Clearly, if we denote the network graph derived from α by G(α), then, for α_1_ < α_2_, G(α_2_) must be a subgraph of G(α_1_); that is, as the α value decreases, additional nodes and edges will be added to the former graphs. Although the networks induced under greater α values are inclined to be more reliable, some important interactions are possibly missed. Table 1 lists the connection degrees of all genes in the constructed gene regulatory networks under different α values and the average connection degrees. The indegrees are presented in parentheses. From the table, we can see that the connectivity of the majority of the nodes is close to each other, and a small number of nodes have relatively low connectivity. An interesting phenomenon is that the upregulated genes are regulated by more other genes than the downregulated genes, while the downregulated genes regulate more other genes than the upregulated genes. This is particularly evident under such mean α values as 0.8 and 0.85. Actually, when α = 0.8, the average number of genes regulated by the downregulated genes is around nine while the average number of genes regulating the downregulated genes is around five. The *P*-value of the t-test of the difference is approximately 0.0142, indicating significance of the difference. In contrast, when α = 0.8, the average number of genes regulated by the upregulated genes is approximately four while the average number of genes regulating the upregulated genes is approximately eight. The *P*-value of the t-test is approximately 0.0177, also suggesting that the difference is significant. When α = 0.85, the *P*-values of the t-test for the downregulated genes and the upregulated genes are 0.0004 and 0.0366, respectively. In general, when α equals 0.8 or 0.85, we reach a more ideal balance between the identified gene-interaction numbers and the reliability of the identified interactions, relative to the other α values. Therefore, the above results revealing the difference in regulatory direction for the two classes of cancer-related genes are meaningful.

**Figure 4.**
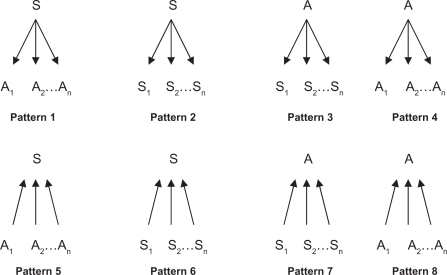
Eight regulatory patterns. **Abbreviations:** S, suppressor; S_i_, the ith suppressor; A, activator; A_i_, the ith activator, i = 1, 2, …, n.

As we know, one common property of biological systems is robustness, which is a consequence of natural selection and facilitates the evolvability of biological systems.^41^–^50^ Robustness enables biological systems to withstand perturbations in the form of various diseases, including cancer. Although the mechanism underlying cancer remains unclear, accumulated evidence has revealed that cancer is caused by genetic perturbations.^51^–^67^ Therefore, biological systems may have evolved to become robust to genetic perturbations to resist the occurrence of cancer.^48^–^50^ Here we refer to upregulated genes in tumor as activators and to downregulated genes as suppressors. We assume eight regulatory patterns, as shown in Figure 4. Pattern 1 represents one suppressor suppressing multi-activators; Pattern 2 represents one suppressor activating multisuppressors; Pattern 3 represents one activator suppressing multi-suppressors; Pattern 4 represents one activator activating multi-activators; Pattern 5 represents one suppressor being suppressed by multiactivators; Pattern 6 represents one suppressor being activated by multi-suppressors; Pattern 7 represents one activator being suppressed by multi-suppressors; and Pattern 8 represents one activator being activated by multi-activators. For robust biological systems, Patterns 1, 2, 6, and 7 should be strong while the others should be weak; that is, the suppressors should function as the inhibitors of tumor as strongly as possible by suppressing more tumor activators and activating more tumor suppressors. In contrast, the activators should function as the enhancers of tumor as weakly as possible by suppressing less tumor suppressors and activating less tumor activators. To prove the conjecture, for every identified gene, we calculate the value of n, which is the number of genes regulating the gene or being regulated by the gene under specific patterns with α = 0.8. We use n to indicate the strength of the patterns. The larger n is, the stronger the corresponding pattern is. Table 2 presents the value of n, suggesting that Patterns 1, 2, 6, and 7 are strong while Patterns 3, 4, 5, and 8 are relatively weak. Here we choose to analyze the network constructed with α = 0.8 on the basis of mainly two considerations: first, we obtain the best classification accuracy when α = 0.8;^34^ second, the sensitivity and specificity of the induced regulatory relations could reach a better balance when α = 0.8 relative to the other α values; that is, a substantial number of comparatively reliable gene regulatory relations can be identified when α = 0.8.

In general, much of a cell’s activity is organized as a network of interacting modules: sets of genes coregulated to respond to different conditions.^68^ Modules constitute the “building blocks” of molecular networks.^49^ The modular organization of molecular networks ensures functionality and robustness of biological systems at some level. To explore the modularity of our colon-cancer-specific gene regulatory networks, we use the Cytoscape plugin MCODE^69^ to analyze the network constructed under α = 0.8. Two significant modules are detected. They are presented in Table 3. The first module is composed of 11 nodes and 66 edges. Its clustering coefficient is 0.6, which is rather high.^70^ The second module is composed of three nodes and three edges, forming a feedforward loop, which is one consensus motif detected in complex networks^71^ including transcriptional regulation networks.^72^ The three nodes represent three upregulated genes, respectively. It possibly indicates that the co-regulations of multiple activators are at least partly, if not completely, responsible for the occurrence of tumor. Further, we use the Cytoscape plugin BiNGO^73^ to perform a GO (Gene Ontology) based enrichment analysis of the two modules (see Table S1 in the Supplementary Materials).

### Network type 2

**Figure 5.**
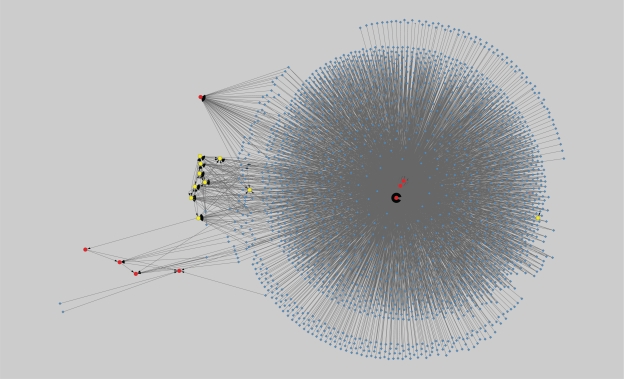
Network Type 2 constructed under α = 0.85.

**Figure 6.**
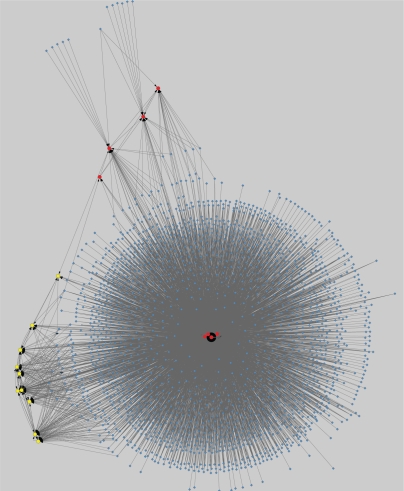
Network Type 2 constructed under α = 0.8.

Network Type 2 exhibits the regulated relations of the identified genes within the genome. We use red circle nodes to represent identified upregulated genes, yellow circle nodes to represent identified downregulated genes, and blue diamond nodes to represent other genes. In addition, we label the nodes representing the identified genes with their gene names, and the other nodes with the attribute number of the corresponding genes in the microarray decision table (the attribute numbers begin from 0). The corresponding regulatory networks when α = 0.85 and 0.8 are shown in Figure 5 and Figure 6, respectively. Similar to the situation in Network Type 1, as the α value decreases, more and more nodes and edges will be added to the former graphs. Table 4 lists the connection degrees of all identified genes in the gene regulatory networks constructed under various α values and the average connection degrees. The indegrees are presented in parentheses.

Regarding Network Type 2, we mainly focus on dissecting the situation that the identified genes are regulated by the other genes. Table 4 shows that the upregulated genes are regulated by more other genes than the downregulated genes. Especially, when α equals 0.85 and 0.8, there are respectively three and four upregulated genes regulated by a large number of other genes so that they form the hubs of extremely dense module subgraphs. To quantitatively analyze the regulation difference between the upregulated genes and the downregulated genes, we respectively calculate the average numbers of genes regulating all upregulated genes and all downregulated genes under various α values as well as their individual averages in whole, and use the t-test to evaluate the significance of the difference. The results presented in Table 5 suggest that the difference is significant when α value is 0.85 and 0.8 with a *P*-value threshold of 0.05. Moreover, the average difference in whole is also significant. As noted above, the choice of analyzing the regulatory relations induced under mean α values is relatively reasonable. Therefore, we can safely conclude that the upregulated genes are more strongly regulated by the other genes than the downregulated genes. It also implies that the upregulated genes instead of the downregulated genes are inclined to form a high degree of centrality in order to play key roles in cancer-specific gene interaction networks. Similar discoveries were made by other authors.^8^,^74^

Further, we use MCODE to analyze the network constructed under α = 0.8. Three significant modules are detected. They are presented in Table 6. It should be noted that the actual clustering coefficients may exceed the presented numbers because we do not take into account the possibility that the non-identified genes are regulated. The results of GO-based enrichment analysis of the three modules are presented in Table S2 in the Supplementary Materials.

## Discussion and Conclusions

The complicated molecular mechanism underlying cancer lies in the perturbations of gene-interaction networks at some level. Therefore, identifying cancer genes and the pathways they control through the networks is a key step toward overcoming cancer. Generally speaking, directed gene regulatory networks reflect the gene interactions more genuinely than undirected gene co-expression networks in that the principal cause-effect relations between genes can be disclosed in directed gene regulatory networks. The present work aims at inferring directed gene regulatory networks under specific disease conditions using formalized rules, which facilitate the interpretability of the inference model. We first identify the genes that are relevant to a specific disease by supervised learning algorithms, and then infer the regulatory relations among the identified genes and their regulated relations by all other genes. Our approach for inferring regulation networks is based on soft computing rules. The reliability of inferred regulation relations depends on the confidence of corresponding rules, which is governed by the controllable parameter α. To ensure sufficiently high reliabilities of the inferred relations, we set a high threshold for α. When analyzing the properties of inferred networks, we often select networks induced with a rational value of α, which contain substantial and reliable regulatory relations.

Our work results in several interesting findings on colon-cancer-specific gene regulatory networks. First, upregulated genes are regulated by more genes than downregulated ones, while downregulated genes regulate more genes than upregulated ones. Second, tumor suppressors suppress tumor activators and activate as many other tumor suppressors as possible. In contrast, tumor activators activate other tumor activators and suppress as few tumor suppressors as possible. This result reflects the robustness of biological systems at some level. For the first finding, we have presented some previous research reports which hold the similar notion. For the second finding, we have given statistical analysis pertinently. Therefore, to a certain extent, the biological results derived based on our assumption are reasonable and relevant. Of course, the reliability of these conclusions needs to be verified with more experimental data.

In terms of our inference rules, A⇒B while A⇐B imply a directed relationship of A toward B. If both A and B are concerned with gene expressions, this relationship can be taken as one kind of regulation relationship rather than simple correlation relationship between gene pairs. In effect, decision rules have been admittedly applied to mining cause-effect relations in machine learning and data mining community. Specifically, the decision logic language (DLL) introduced by Pawlak^75^ gives the formal definition of decision rules, indicative of the cause-effect relationship derived in decision rules.^34^

Further, according to our inference logic, the fact that from the up-regulation of gene A, we can infer the up-regulation of gene B, and from the down-regulation of A, we can infer the down-regulation of B (but not the reverse) means that the expression of gene A can determine the expression of gene B (while the expression of gene B cannot determine the expression of gene A). From this correlation, we can infer the regulation direction, indicating that A regulates B. Thus, the inferred gene-to-gene interaction networks are directed gene regulatory networks more than simple co-expression networks. It should be noted that our directed gene regulatory relations refer to one kind of wide interactions between gene pairs such as the upstream and downstream relations in a signaling pathway, not necessarily implying physical interactions or direct regulations between them. Certainly, we agree that the use of steady gene expression data gives rise to limitations in inference of directed gene regulatory networks, and if perturbation data or time-series data are used in network inference, the inferred pair-wise regulation relations could be more convincing. This is our next study objective.

Our method belongs to the rule-based network inference. In this point, it is similar to decision tree. However, essentially differing from decision tree, our gene regulatory relations are induced by decision rules, which are based on the subset (set inclusion) relations and well formalized in the DLL. In addition, although our soft computing rule resembles to probabilistic score thereby demonstrating the reliability of our inference rules, soft computing approach is essentially different from probability theory in that soft computing exploits the given tolerance of imprecision, partial truth, and uncertainty for a particular problem, making it to model and analyze complex systems in a more flexible and robust manner and finally give useful answers. Soft computing has the major advantages in inductive reasoning and uncertain reasoning.

## Materials and Methods

### Dataset

The microarray dataset we study is the Colon Cancer dataset,^76^ which contains 62 samples collected from colon cancer patients. Among them, 40 tumor biopsies are from tumors and 22 normal biopsies are from healthy parts of the colons of the same patients. Each sample is described by 2000 genes. In our previous work,^34^ we identified 21 genes or ESTs, each of which possesses fairly good classification performance. In this work, we choose to analyze 18 definitely annotated genes out of them, which include DES, MYL9, CSRP1, IL8, S100A11, ACTA2, HSPD1, HNRNPA1, SPARCL1, DARS, KCNMB1, MGP, SLC2A4, myosin, TPM3, SRPK1, IPL1, and PCBD1.

The microarray dataset studied by our methodology is organized in the form of decision tables. One decision table can be represented by *S* = (*U*, *A* = *C* ∪ *D*), where *U* is the set of samples, *C* the condition attribute set, and *D* the decision attribute set. Table 7 is the decision table representing the Colon Cancer microarray dataset. In the decision table, there are 62 samples, 2000 condition attributes, and one decision attribute. Every sample is assigned to one class label: Tumor or Normal.

In the decision table, we define a function *I_a_* that maps a member (sample) of *U* to the value of the member on the attribute *a* (*a ∈A*), and an equivalence relation *R*(*A*’) induced by the attribute subset *A*’ ⊆*A*, as follows: for *x*, *y∈U*, *xR*(*A*’)*y* if and only if *I_a_*(*x*) = *I_a_*(*y*) for each *a∈A*’.^34^

### α Depended Degree, Decision Rules, and Learning Algorithm

In,^34^ we identify one high class-discrimination feature based on the α depended degree, which is a generalization of the depended degree proposed in rough sets.^77^ Here we restate the concept briefly. The α *depended degree* of condition subset *P* by decision attribute set *D* is defined by:

$$\gamma_{P}(D,\alpha )=\frac{\left|{\text{POS}}_{P}(D,\alpha )\right|}{\left|U\right|},$$

where 0 ≤ *α* ≤ 1,

$$|{\text{POS}}_{P}(D,\alpha )|=|\underset{X\in U/R(D)}{\cup }\;\text{pos}(P,X,\alpha )|$$

and *pos*(*P*, *X* ,α)= ∪ {*Y ∈ U*/*R*(*P*) | |*Y* ∩ *X* |/|*Y* |≥ α}. Here |*| denotes the size of set * and *U/R*(•) denotes the set of equivalence classes induced by the equivalence relation *R*(•). The depended degree is a specific case of the *α* depended degree when α *=* 1.^34^

In,^34^ we create classifiers based on decision rules. One decision rule in the form of “*A* ⇒ *B*” indicates that “if *A*, then *B*,” where *A* is the description of condition attributes and *B,* the description of decision attributes. The *confidence* of a decision rule *A* ⇒ *B* is defined as follows:

$$\text{confidence}(A\Rightarrow B)=\frac{\text{support}(A\wedge B)}{\text{support}(A)},$$

where *support*(*A*) denotes the proportion of samples satisfying *A* and *support*(*A* ∧ *B*) denotes the proportion of samples satisfying *A* and *B* simultaneously. The confidence of a decision rule indicates the reliability of the rule.

In,^34^ for each determined *α* value, we select only the genes with *γ_P_*(*D*,*α*) = 1 to build decision rules. Suppose *g* is one of the selected genes and *U* is the sample set. *U/R*(*g*) = {*c*_1_(*g*), *c*_2_(*g*), …, *c_n_*(*g*)} represents the set of the equivalence class of samples induced by *R*(*g*). Two samples, *s*_1_ and *s*_2_, belong to the same equivalence class of *U/R*(*g*) if and only if they have the same value on *g*. In addition, we represent the set of the equivalence class of samples induced by *R*(*D*) as *U/R*(*D*) = {*d*_1_(*D*), *d*_2_(*D*), …, *d_m_*(*D*)}, where *D* is the decision attribute. Likewise, two samples, *s*_1_ and *s*_2_, belong to the same equivalence class of *U/R*(*D*) if and only if they have the same value on *D*. For each *c_i_*(*g*) (*i* = 1, 2, …, *n*), if there exists some value of *d_j_*(*D*) (*j*∈{1, 2, …, *m*}), satisfying *c_i_*(*g*)⊆*d_j_*(*D*) in light of the depended degree or |*c_i_*(*g*)∩*d_j_*(*D*)|/|*c_i_*(*g*)|≥*α* in light of the *α* depended degree, we then generate the following decision rule: *A*(*c_i_*(*g*)) ⇒ *B*(*d_j_*(*D*)), where *A*(*c_i_*(*g*)) is the formula describing the sample set *c_i_*(*g*) by the *g* value, and *B*(*d_j_*(*D*)) is the formula describing the sample set *d_j_*(*D*) by the class value. We ensure sufficient reliability of the derived decision rules by setting a high threshold for the *α* value.

Because our method is suitable for handling discrete data, we discretize the original microarray dataset decision table before carrying out the learning algorithm. We use the entropy-based discretization method^78^ and implement the discretization in the Weka package.^79^ Table 8 is the discretized decision table of Table 7. From Table 8, we can infer that Gene 1 and Gene 2000 cannot distinguish different classes, while Gene 249 can distinguish different classes by two decision rules: if the expression level of Gene 249 in one sample is not greater than 1696.2275, then the sample is Tumor (89% confidence); otherwise, the sample is Normal (86% confidence); that is, if Gene 249 is downregulated in one sample, then the sample is Tumor; if Gene 249 is upregulated in one sample, then the sample is Normal. Using the two rules, we achieve 84% leave-one-out cross-validation (LOOCV) accuracy. Among the aforementioned 18 genes, DES, MYL9, CSRP1, ACTA2, SPARCL1, KCNMB1, MGP, SLC2A4, myosin, and TPM3 belong to downregulated genes in Tumor, while IL8, S100A11, HSPD1, HNRNPA1, DARS, SRPK1, IPL1, and PCBD1 belong to upregulated genes in Tumor.

### Inference of gene regulatory network

If the decision attribute is one gene instead of the class, then we can induce the decision rules inferring regulatory relations among distinct genes. For example, if we substitute “Gene 249” for “Class label” in Table 7, that is, we regard Gene 249 as the decision attribute, which has two distinct values: upregulation and downregulation, we obtain Table 9.

Likewise, we implement the discretization of Table 9 to obtain Table 10. Applying the same learning algorithm to Table 10, we can induce the decision rules linking Gene 245 to Gene 249: if the expression level of Gene 245 in one sample is not greater than 1048.3779, then Gene 249 is downregulated (96% confidence); otherwise, Gene 249 is upregulated (100% confidence). In other words, if Gene 245 is downregulated, then Gene 249 is downregulated; if Gene 245 is upregulated, then Gene 249 is upregulated. They are not necessarily true in reverse. Therefore, we infer a directed regulatory relation of Gene 245 to Gene 249, which is positive.

In the same way, we regard each of the 18 identified genes as the decision attribute in turn, and infer the regulatory relations that the other genes exert on them. We infer those networks with α value equal to 1, 0.95, 0.9, 0.85, 0.8, 0.75, or 0.7.

## Supplemental Materials

**Table S1.** GO terms significantly enriched with two modules in Network Type 1 (α = 0.8).

**GO/Module**	**Category**	**GO-ID**	**Description**	***P-*value**
1	Molecular function	48306	Calcium-dependent protein binding	0.00003
2	Biological process	42221	Response to chemical stimulus	0.005
	Molecular function	5524	ATP binding	0.02
		32559	Adenyl ribonucleotide binding	0.02
		30554	Adenyl nucleotide binding	0.02

GO terms shared by more than one gene with *P* ≤ 0.05 are identified.

**Table S2.** GO terms significantly enriched with three modules in Network Type 2 (α = 0.8).

**GO/Module**	**Category**	**GO-ID**	**Description**	***P-*value**
1	Biological process	51239	Regulation of multicellular organismal process	0.001
		51170	Nuclear import	0.002
		51098	Regulation of binding	0.003
		45941	Positive regulation of transcription	0.003
		10628	Positive regulation of gene expression	0.003
		45935	Positive regulation of nucleobase, nucleoside, nucleotide and nucleic acid metabolic process	0.003
		10557	Positive regulation of macromolecule biosynthetic process	0.004
		9891	Positive regulation of biosynthetic process	0.005
		6913	Nucleocytoplasmic transport	0.005
		51169	Nuclear transport	0.005
		10604	Positive regulation of macromolecule metabolic process	0.006
	Molecular function	48306	Calcium-dependent protein binding	0.00007
2	Cellular component	44449	Contractile fiber part	0.0004
		43292	Contractile fiber	0.0004
3	Biological process	6395	RNA Splicing	0.001
		6394	RNA processing	0.003
	Molecular function	166	Nucleotide binding	0.002

GO terms shared by more than one gene with *P* ≤ 0.05 are identified.

## Figures and Tables

**Table 1.** Connection degrees of identified genes in Network Type 1.

**Gene/α**	**1**	**0.95**	**0.9**	**0.85**	**0.8**	**0.75**	**0.7**	**Average**
DES	0	1 (1)	2 (2)	10 (5)	14 (6)	17 (8)	22 (13)	9 (5)
MYL9	0	0	2 (1)	9 (3)	12 (4)	24 (13)	27 (15)	12 (5)
CSRP1	0	1 (0)	2 (0)	10 (4)	20 (12)	23 (13)	27 (16)	12 (6)
ACTA2	0	0	4 (3)	9 (3)	12 (3)	18 (5)	27 (13)	10 (4)
SPARCL1	0	1 (1)	4 (3)	10 (3)	15 (7)	20 (8)	27 (15)	11 (5)
KCNMB1	0	0	6 (3)	13 (4)	14 (4)	29 (15)	29 (15)	13 (6)
Mgp	0	0	4 (0)	11 (2)	16 (4)	26 (13)	27 (13)	12 (5)
SLC2A4	0	0	1 (0)	5 (1)	12 (2)	24 (11)	25 (11)	10 (4)
myosin	0	0	0	3 (0)	7 (3)	15 (10)	19 (14)	6 (4)
TPM3	0	1 (0)	4 (2)	13 (6)	18 (9)	22 (9)	22 (9)	11 (5)
**IL8**	0	0	1 (1)	3 (1)	6 (2)	21 (9)	22 (9)	8 (3)
**S100A11**	0	1 (0)	2 (0)	14 (12)	16 (13)	21 (13)	26 (15)	11 (8)
**HSPD1**	0	0	0	3 (2)	7 (5)	14 (6)	15 (6)	6 (3)
**HNRNPA1**	0	0	2 (2)	5 (3)	17 (12)	22 (13)	27 (13)	10 (6)
**DARS**	0	0	0	3 (1)	7 (3)	13 (5)	18 (5)	6 (2)
**SRPK1**	0	0	0	1 (1)	5 (3)	10 (4)	15 (5)	4 (2)
**IPL1**	0	0	0	13 (11)	14 (11)	20 (12)	25 (13)	10 (7)
**PCBD1**	0	1 (1)	2 (1)	14 (13)	17 (12)	21 (12)	26 (13)	12 (7)

The upregulated genes are formatted in boldface in table 1, 2 and 4.

**Table 2.** Values of n for eight regulatory patterns detected when α = 0.8.

**Pattern/Gene**	**1**	**2**	**3**	**4**	**5**	**6**	**7**	**8**
DES	4	4			0	6		
MYL9	4	4			0	4		
CSRP1	4	4			5	7		
ACTA2	4	5			0	3		
SPARCL1	4	4			0	7		
KCNMB1	4	6			0	4		
Mgp	4	8			0	4		
SLC2A4	4	6			0	2		
myosin	3	1			1	2		
TPM3	4	5			0	9		
**IL8**			1	3			1	1
**S100A11**			1	2			10	3
**HSPD1**			1	1			0	5
**HNRNPA1**			0	5			8	4
**DARS**			1	3			0	3
**SRPK1**			1	1			0	3
**IPL1**			1	2			10	1
**PCBD1**			1	4			10	2

**Table 3.** Properties of two modules detected in network Type 1 with α = 0.8.

**Module/Property**	**1**	**2**
Genes contained in the module	PCBD1, TPM3, S100A11, SPARCL1, HNRNPA1, KCNMB1, ACTA2, IPL1, Mgp, SLC2A4, CSRP1	HSPD1, IL8, DARS
Node number	11	3
Edge number	66	3
Clustering coefficient	0.6	0.5
Upregulated genes	PCBD1, S100A11, HNRNPA1, IPL1	HSPD1, IL8, DARS
Downregulated genes	TPM3, SPARCL1, KCNMB1, ACTA2, Mgp, SLC2A4, CSRP1	N/A

“N/A” indicates that there is no related gene contained in the corresponding modules.

**Table 4.** Connection degrees of identified genes in Network Type 2.

**Gene/α**	**1**	**0.95**	**0.9**	**0.85**	**0.8**	**Average**
DES	0	2 (2)	4 (3)	15 (10)	33 (25)	11 (8)
MYL9	0	0	7 (6)	22 (17)	40 (33)	14 (11)
CSRP1	1 (1)	3 (2)	3 (2)	9 (5)	18 (13)	7 (5)
ACTA2	0	0	11 (10)	28 (22)	30 (22)	14 (11)
SPARCL1	1 (1)	2 (2)	6 (5)	20 (13)	36 (28)	13 (10)
KCNMB1	0	0	13 (10)	24 (15)	40 (29)	15 (9)
Mgp	0	0	12 (8)	30 (21)	47 (36)	18 (13)
SLC2A4	0	0	10 (9)	27 (23)	63 (54)	20 (17)
myosin	0	0	0	3 (1)	10 (6)	3 (1)
TPM3	0	0	6 (4)	24 (18)	53 (45)	17 (13)
**IL8**	0	0	1 (1)	2 (1)	8 (4)	2 (1)
**S100A11**	0	4 (3)	67 (64)	1369 (1367)	1401 (1399)	568 (567)
**HSPD1**	0	0	0	8 (7)	28 (27)	7 (7)
**HNRNPA1**	0	0	6 (6)	57 (55)	1752 (1747)	363 (362)
**DARS**	0	0	0	7 (5)	37 (33)	9 (8)
**SRPK1**	0	0	2 (2)	9 (9)	22 (21)	7 (6)
**IPL1**	0	6 (6)	57 (57)	1772 (1770)	1787 (1785)	724 (724)
**PCBD1**	0	13 (13)	84 (83)	1569 (1568)	1595 (1591)	652 (651)

**Table 5.** Contrast in regulatory circumstances of two groups of genes.

**Statistics/α**	**0.95**	**0.9**	**0.85**	**0.8**	**Average**
Average number of genes regulating upregulated genes	2.75	26.625	597.75	825.875	290.75
Average number of genes regulating downregulated genes	0.6	5.7	14.5	29.1	9.8
*P*-value (t-test)	0.1199	0.0679	0.0407	0.0178	0.0214

**Table 6.** Properties of three modules detected in Network Type 2 with α = 0.8.

**Module/α Property**	**1**	**2**	**3**
Genes contained in the module	PCBD1, S100A11, Mgp, SPARCL1, SLC2A4, IPL1, HNRNPA1, TPM3, BCL3, MAOB, SDC2, SRF, PRDX6, VIP, CALD1, DELTA-CRYSTALLIN ENHANCER BINDING FACTOR	DES, KCNMB1, MYL9, ACTA2, CEBPD, CCND3, SRF	HSPD1, SRPK1, HNRNPM
Nodes number	16	7	3
Edges number	88	14	4
Clustering coefficient	0.37	0.33	0.25
Upregulated genes	PCBD1, S100A11, IPL1, HNRNPA1, SDC2	N/A	HSPD1, SRPK1, HNRNPM
Downregulated genes	Mgp, SPARCL1, SLC2A4, TPM3, SRF, BCL3, MAOB, PRDX6, VIP, CALD1, DELTA-CRYSTALLIN ENHANCER BINDING FACTOR	DES, MYL9, ACTA2, KCNMB1, CCND3, CEBPD, SRF	N/A

“N/A” indicates that there is no related gene contained in the corresponding modules.

**Table 7.** Colon cancer microarray dataset decision table.

**Sample**	**Condition attribute (gene)**					**Decision attribute (class)**
	**Gene 1**	**…**	**Gene 249**	**…**	**Gene 2000**	**Class label**
1	8589.4163	…	500.425	…	28.70125	Tumor
2	9164.2537	…	335.69	…	16.77375	Normal
…	…	…	…	…	…	…
61	6234.6225	…	272.92875	…	23.265	Tumor
62	7472.01	…	2699.1925	…	39.63125	Normal

**Table 8.** Discretized colon cancer microarray dataset decision table.

**Sample**	**Condition attribute (gene)**					**Decision attribute (class)**
	**Gene 1**	**…**	**Gene 249**	**…**	**Gene 2000**	**Class label**
1	‘All’	…	‘(-inf-1696.2275)’	…	‘All’	Tumor
2	‘All’	…	‘(1696.2275-inf)’	…	‘All’	Normal
…	…	…	…	…	…	…
61	‘All’	…	‘(-inf-1696.2275)’	…	‘All’	Tumor
62	‘All’	…	‘(1696.2275-inf)’	…	‘All’	Normal

“ ‘All’ ” indicates that one gene has the same value in all samples; “ ‘(-inf-x)’ ” indicates “<=x”; “ ‘(x-inf)’ ” indicates “>x”.

**Table 9.** Colon cancer microarray dataset decision table.

**Sample**	**Condition attribute (gene)**					**Decision attribute (class)**
	**Gene 1**	**…**	**Gene 245**	**…**	**Gene 2000**	**Gene 249**
1	8589.4163	…	475.27885	…	28.70125	Downregulation
2	9164.2537	…	1648.4596	…	16.77375	Upregulation
…	…	…	…	…	…	…
61	6234.6225	…	191.33846	…	23.265	Downregulation
62	7472.01	…	1240.5846	…	39.63125	Upregulation

**Table 10.** Discretized decision table of Table 9.

**Sample**	**Condition attribute (gene)**					**Decision attribute (class)**
	**Gene 1**	**…**	**Gene 245**	**…**	**Gene 2000**	**Gene 249**
1	‘All’	…	‘(-inf-1048.3779)’	…	‘All’	Downregulation
2	‘All’	…	‘(1048.3779-inf)’	…	‘All’	Upregulation
…	…	…	…	…	…	…
61	‘All’	…	‘(-inf-1048.3779)’	…	‘All’	Downregulation
62	‘All’	…	‘(1048.3779-inf)’	…	‘All’	Upregulation
